# Supplementary material for: Cross-regulation of viral kinases with cyclin A secures shutoff of host DNA synthesis
Source: Nat Commun. 2020 Sep 24;11:4845. doi: 10.1038/s41467-020-18542-1 (PMC7518283; doi:10.1038/s41467-020-18542-1)
Supplement: Supplementary file 1 — Supplementary Information [file 41467_2020_18542_MOESM1_ESM.pdf]

Supplementary Information (SI)

for

Cross-regulation of viral kinases with cyclin A secures shutoff of host DNA synthesis

Bogdanow et al.

Correspondence to Lüder Wiebusch (email: lueder.wiebusch@charite.de)

Supplementary Figure 1

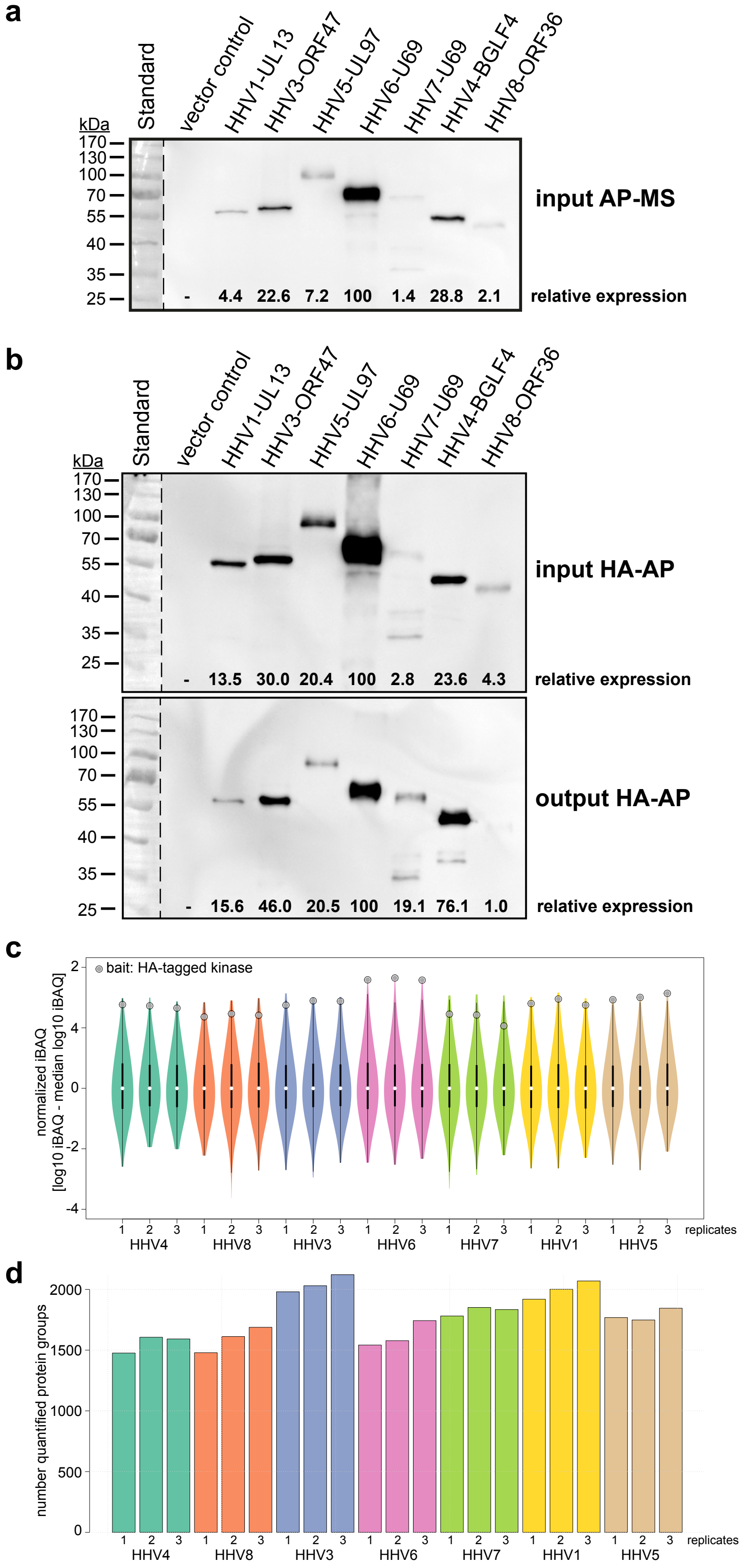

Supplementary Fig. 1. Quality controls of the protein-protein interactome of HHV kinases. (a-b) HEK293T cells were transfected with the indicated expression plasmids. Bait abundance levels in the extracts **(a, b top)** and the eluates from affinity purification columns (b, lower) were analyzed by quantitative imaging of anti-HA immunoblots. **(c)** Violin plot showing normalized iBAQvalues of all proteins quantified in the eluates of CHPK-IPs. SILAC channels of empty vector controls are not depicted. Violin plots are combined density and boxplots. Center: median, thick lines: upper and lower quartiles, thin lines: 1.5x interquartile range. **(d)** The number of quantified protein groups is depicted for the indicated AP-MS experiments. The barplots represent single values. **(c, d)** All n=3 replicates are depicted individually.

Supplementary Figure 2

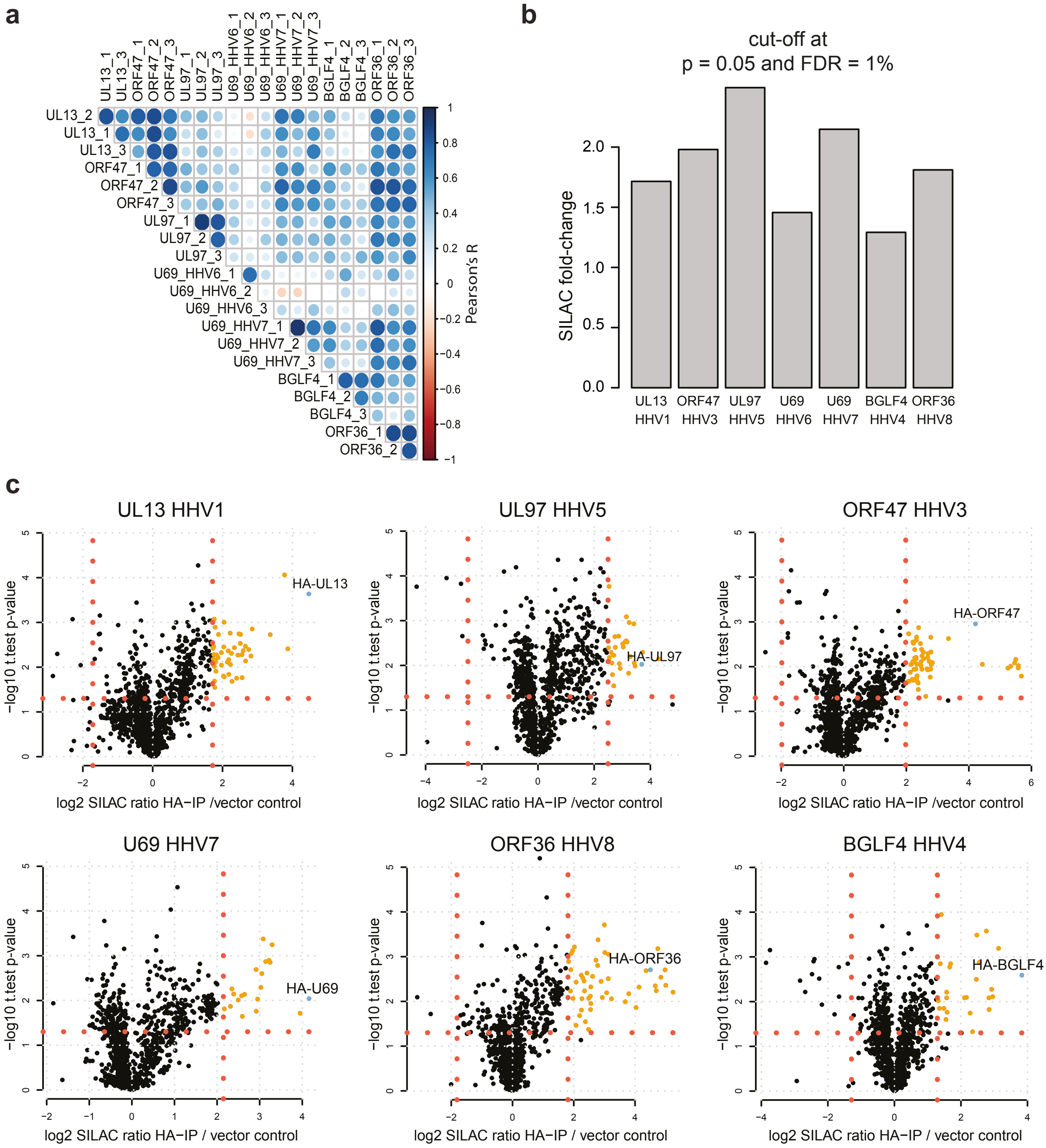

Supplementary Fig. 2. Data analysis of the protein-protein Interactome of HHV kinases. (a) Pearson's correlation coefficients (Pearson's R) comparing individual AP-MS experiments and replicates. **(b)** SILAC fold-change cut-offs when a t-test p-value of 0.05 and a FDR at 1% was used. **(c)** Individual volcano plots for IPs with six different herpesviral kinases. Candidate interaction partners above the fold-change and p-value cut-offs (indicated by red dotted lines) are highlighted orange.

Supplementary Figure 3

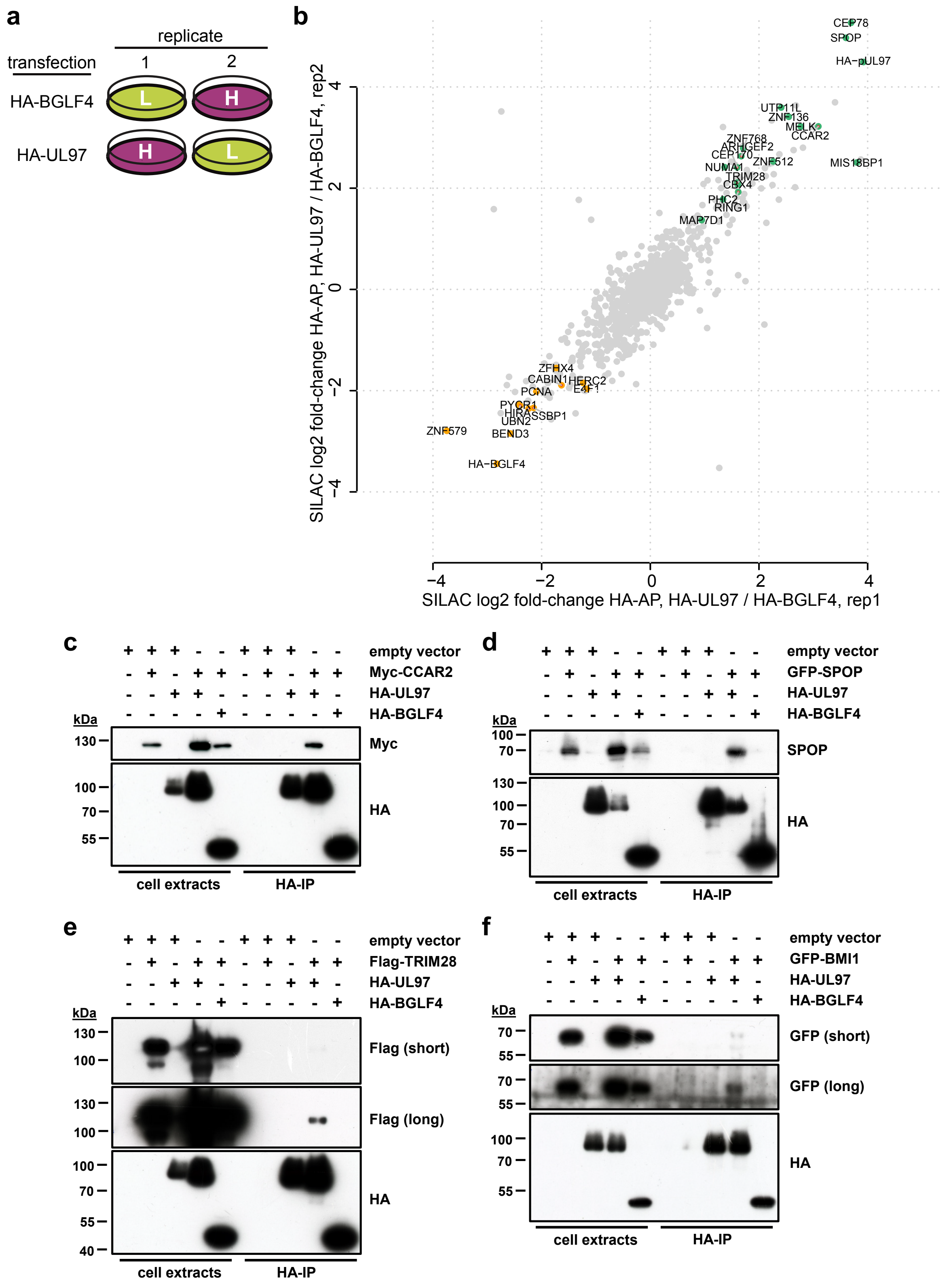

Supplementary Fig. 3. Validation of selected unique CHPK interactors. (a) Experimental design. Expression plasmids encoding HA-tagged versions of HHV5-UL97 and HHV4-BGLF4 were transfected into full SILAC-labeled (Light and Heavy) HEK293T cells. The experiment was designed in label-swap duplicates (n=2). **(b)** Scatterplot of AP-MS SILAC log2 fold-changes directly comparing both CHPKs for both replicates. Highlighted are the baits and unique interactors to HHV5 (green dots, see cluster in Fig.1c labeled as “SUMOylation of transcription cofactors”, “Polycomb repressive complex 1”) and HHV4 CHPKs (orange dots, see cluster in Fig. 1c labeled as “Chromatin silencing”, “DNA replication”). **(c-f)** Validation of select interactors to HHV5-UL97. The indicated expression plasmids were (co-)transfected into HEK-293T cells and co-IPs were performed by precipitating the HA-tagged kinases. Bait and prey proteins were detected by immunoblot analysis.

Supplementary Figure 4

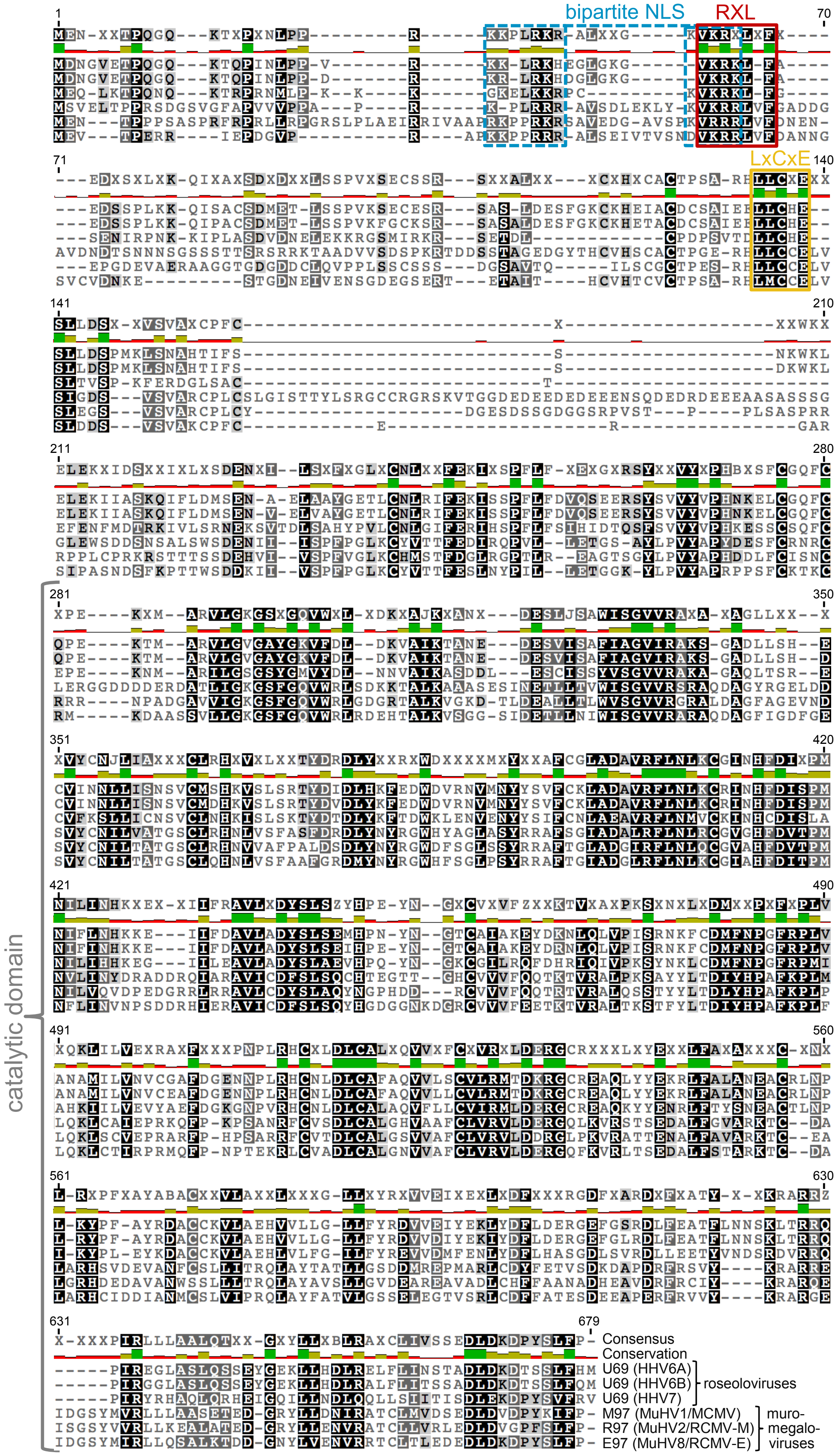

Supplementary Fig. 4. Multiple sequence alignment of β-herpesviral kinases. Multiple sequence alignment was performed including kinases of roseolo- and muromegaloviruses. The conserved catalytic domain is indicated by brackets. The conserved bipartite NLS (blue), the RXL/Cy motif (red) and LXCXE motif (yellow) localize to the otherwise less-conserved N-terminal domain.

Supplementary Figure 5

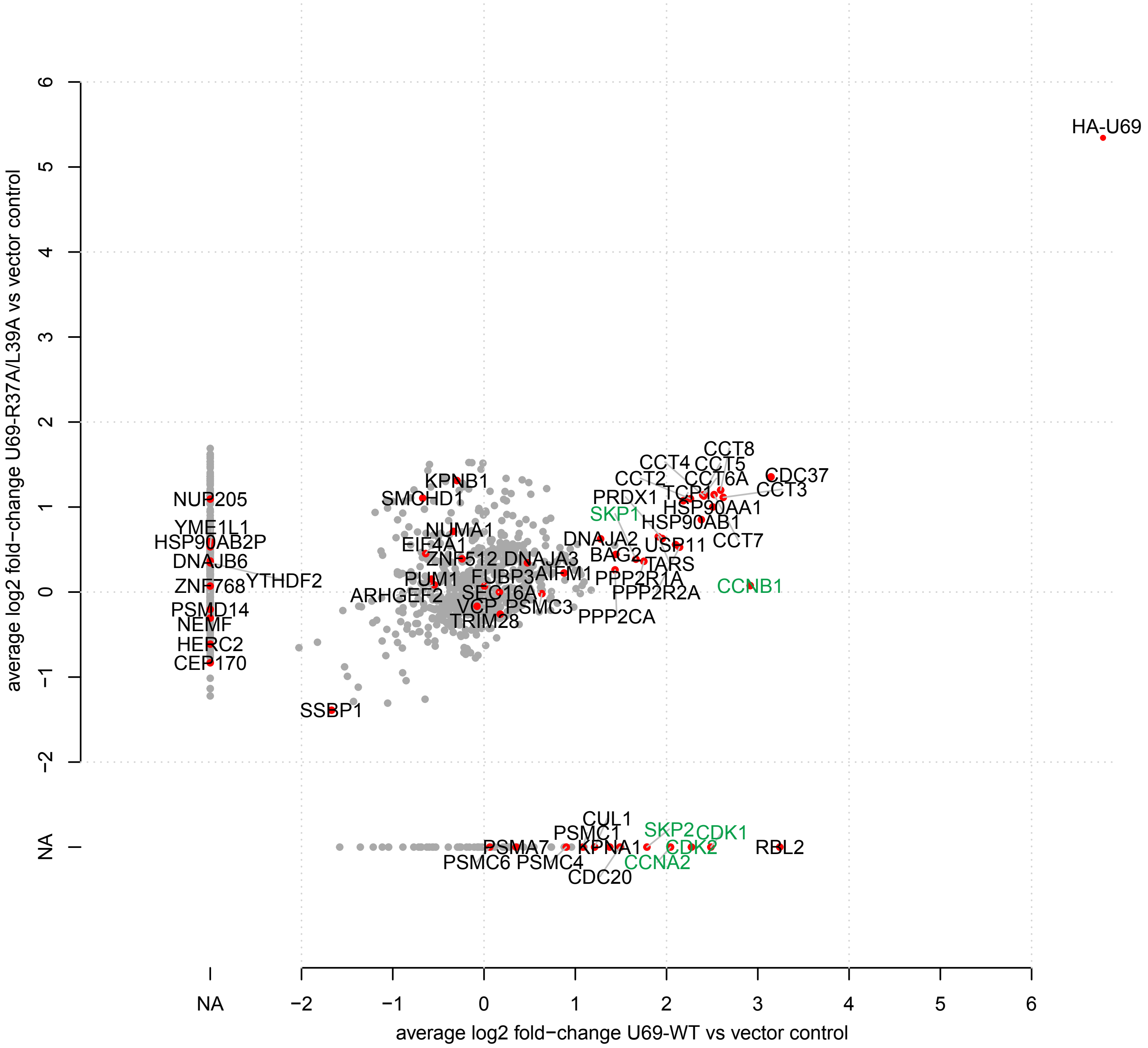

Supplementary Fig. 5. The RXL/Cy-motif assembles higher-order Cyclin-CDK complexes. SILAC-labeled HEK293-T cells were transfected with WT or R37A/L39A mutant versions of HHV6-U69 in label-swap duplicates (n=2). 24 h post transfection samples were subjected to AP-MS and shotgun proteomics. The enrichment of proteins co-purifying with WT versus vector control was plotted against the enrichment of mutant versus vector control SILAC log2 fold-changes. Gene names are given for proteins that are interacting with any of the herpesviral kinases (Supplementary Data 1). Cyclins, CDKs and associated factors are highlighted green. Data represent means of n=2 replicates.

Supplementary Figure 6

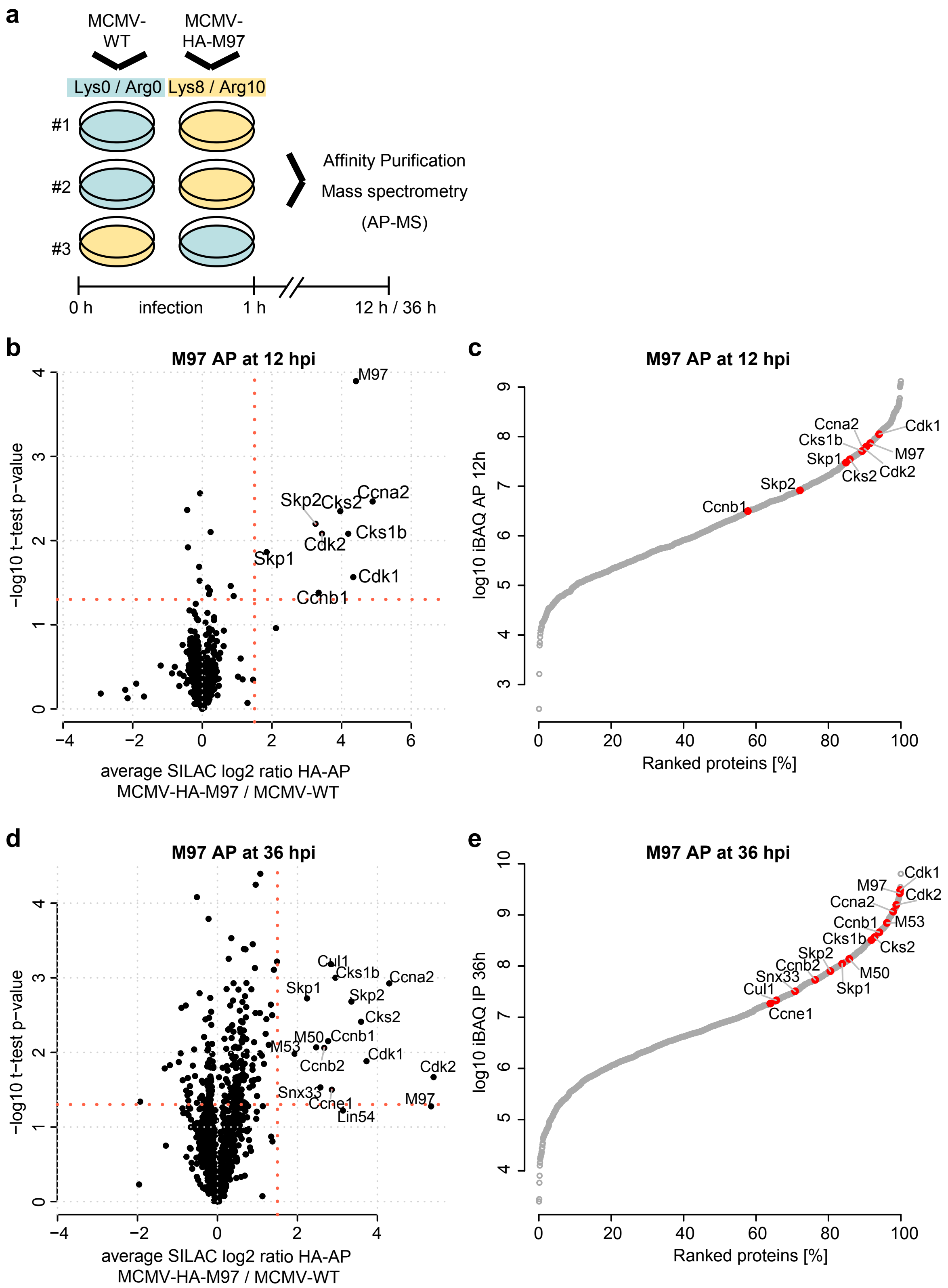

Supplementary Fig. 6. The time-resolved interactome of M97 during infection. (a) Experimental setup. SILAC heavy and light labeled cells were infected with MCMV-WT virus or a MCMV strain that expresses HA-tagged M97. Samples were subjected to AP-MS. The experiment was designed in triplicates (n=3). **(b, d)** The SILAC ratio MCMV-HA-M97 / MCMV-WT served to discriminate interactors of M97 from background binders. The average (n=3 replicates) SILAC fold change and the p-value of a one sample two-sided t-test of the three biological replicates is depicted for the AP at 12 **(b)** and 36 **(d)** hours post infection. **(c, e)** iBAQ values were calculated in HA-M97 MS samples at 12 h **(c)** and 36 h **(e)** post infection. Specific interactors at 12 and 36 h post infection are highlighted in red. The sum of n=3 replicates is depicted. iBAQ values correlate with the molar amount of proteins in a sample and thus give estimates on the stoichiometry between individual protein complex members.

Supplementary Figure 7

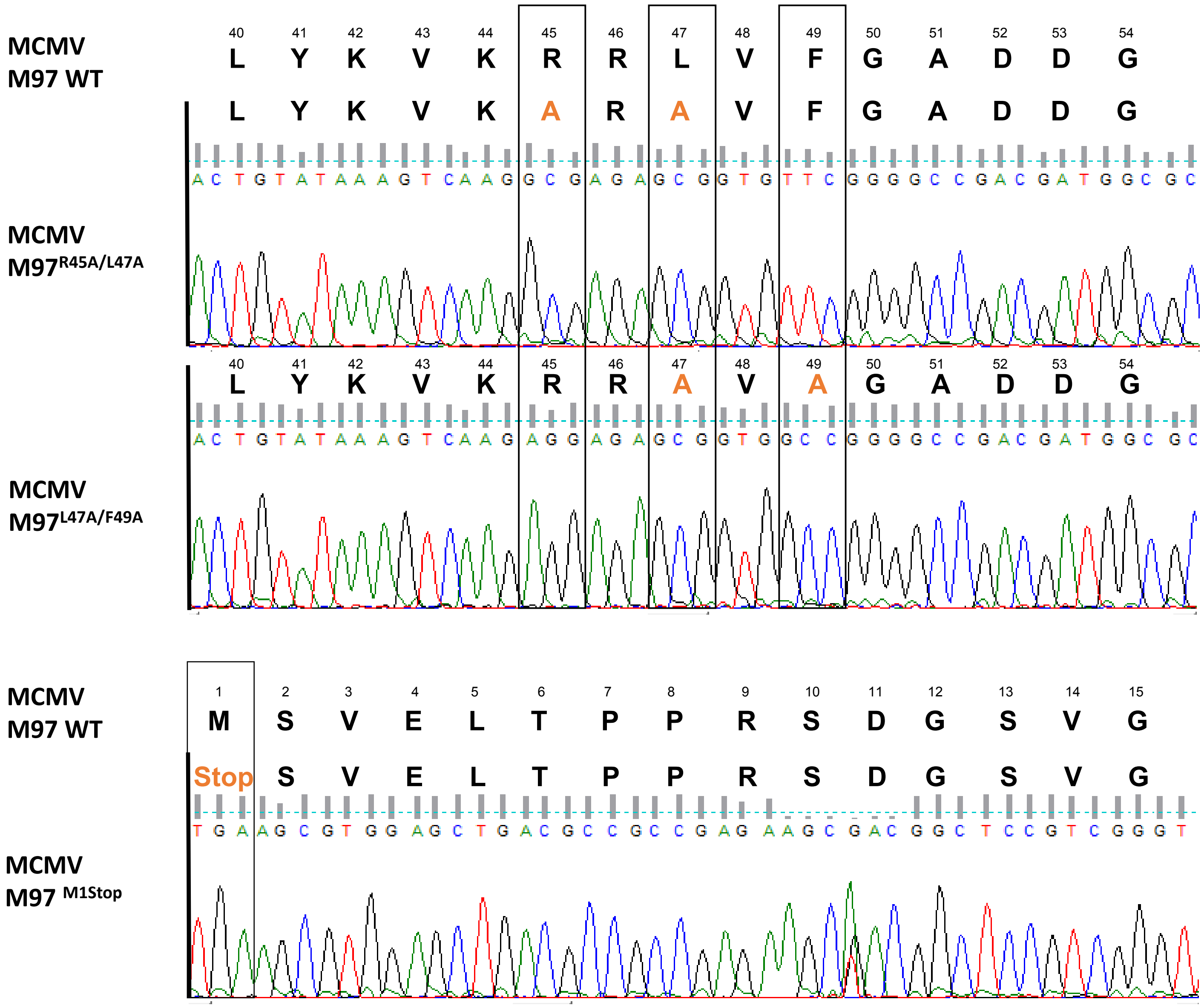

Supplementary Fig. 7. Sanger Sequencing of mutated MCMV-strains. DNA-sequence chromatograms resulting from Sanger sequencing of the mutated M97 gene region from the indicated recombinant viruses. The amino acids marked in orange highlight the introduced mutations.

Supplementary Figure 8

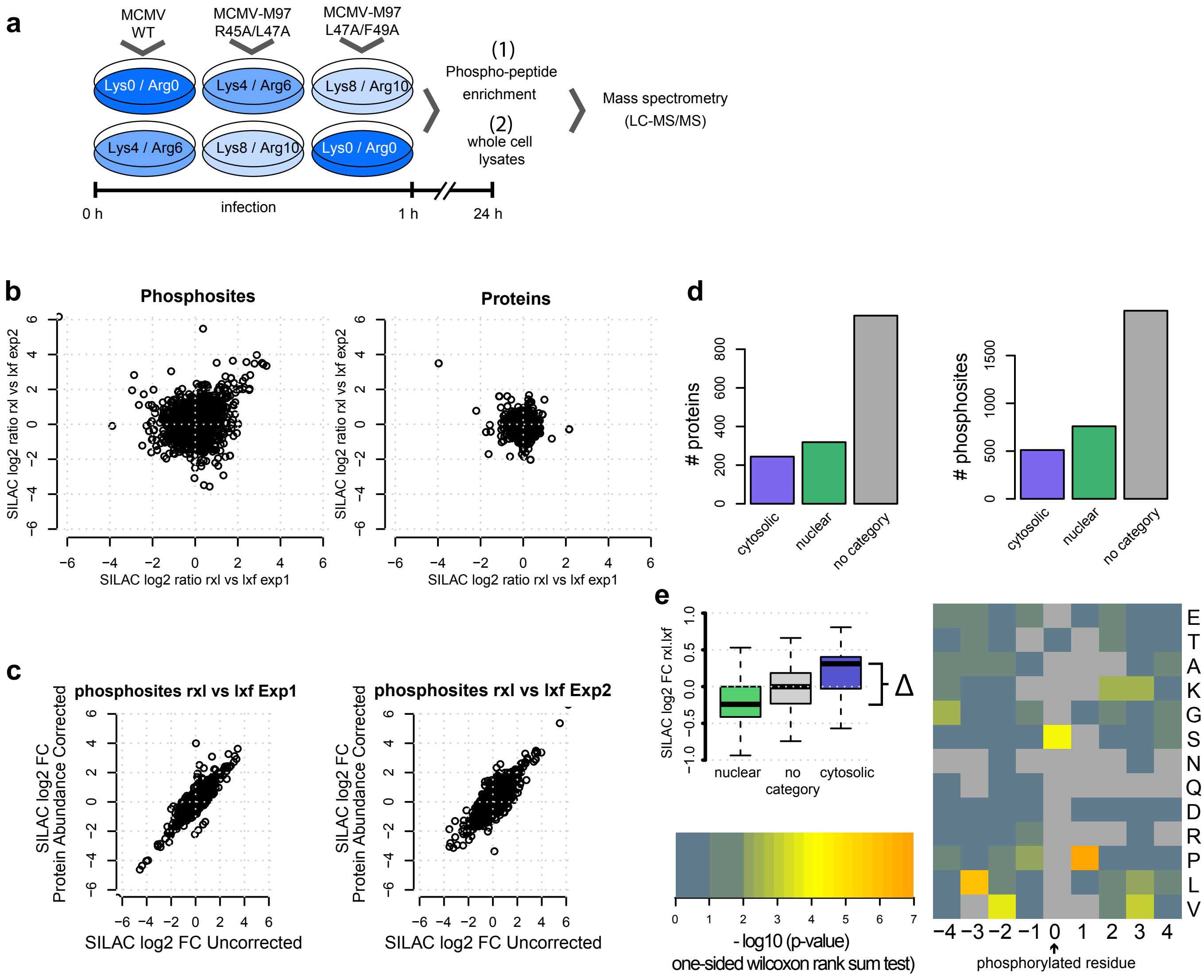

Supplementary Fig. 8. Differences in subcellular phosphoproteomic profiles upon infection with M97 mutant viruses. (a) Experimental setup. SILAC Heavy, Medium and Light labeled cells were infected with MCMV-WT, M97-R45A/L47A or M97-L47A/F49A viruses in label-swap duplicates (n=2). At 24 h post infection cells were either subjected to a phosphoproteomics workflow or whole proteome analysis. Note that only the direct SILAC ratio M97-R45A/L47A / M97-L47A/F49A was used for further analysis. **(b)** Quantified phosphosites and proteins in both replicates in the SILAC comparison M97-R45A/L47A versus M97-L47A/F49A. **(c)** Quantified phosphosites were corrected by the SILAC fold-change of the respective source protein for both replicates. **(d)** Quantified proteins (left panel) and phosphosites (right panel) were classified as cytosolic, nuclear or "no category" (no clear nuclear or cytosolic GO annotation). The number of proteins or phosphosites that were classified in at least one of the replicates is given (single values). **(e)** Global comparison of phosphosites on nuclear proteins with phosphosites on cytosolic proteins by screening any possible amino acid flanking the phosphorylated residue. Upper left panel: Schematic depiction for the calculation of significance estimates (see also Figure 4c). A p-value was calculated to assess the statistical significance of nuclear to cytosolic sites (one-sided Wilcoxon rank sum test). Right panel: Log10 p-values of each amino acid in the region -4 to +4 flanking a Serine or Threonine (position: 0) are depicted. Comparisons were not considered when there were below 19 phosphosites for nuclear and cytosolic proteins (highlighted in grey). We found significant differences for phosphorylated Serines, followed by Prolines at position +1, Lysines at positions +2 and +3 as well as hydrophobic residues at positions -2/-3/+3. (**c-e)** Data represent the averages of n=2 replicates.

Supplementary Figure 9

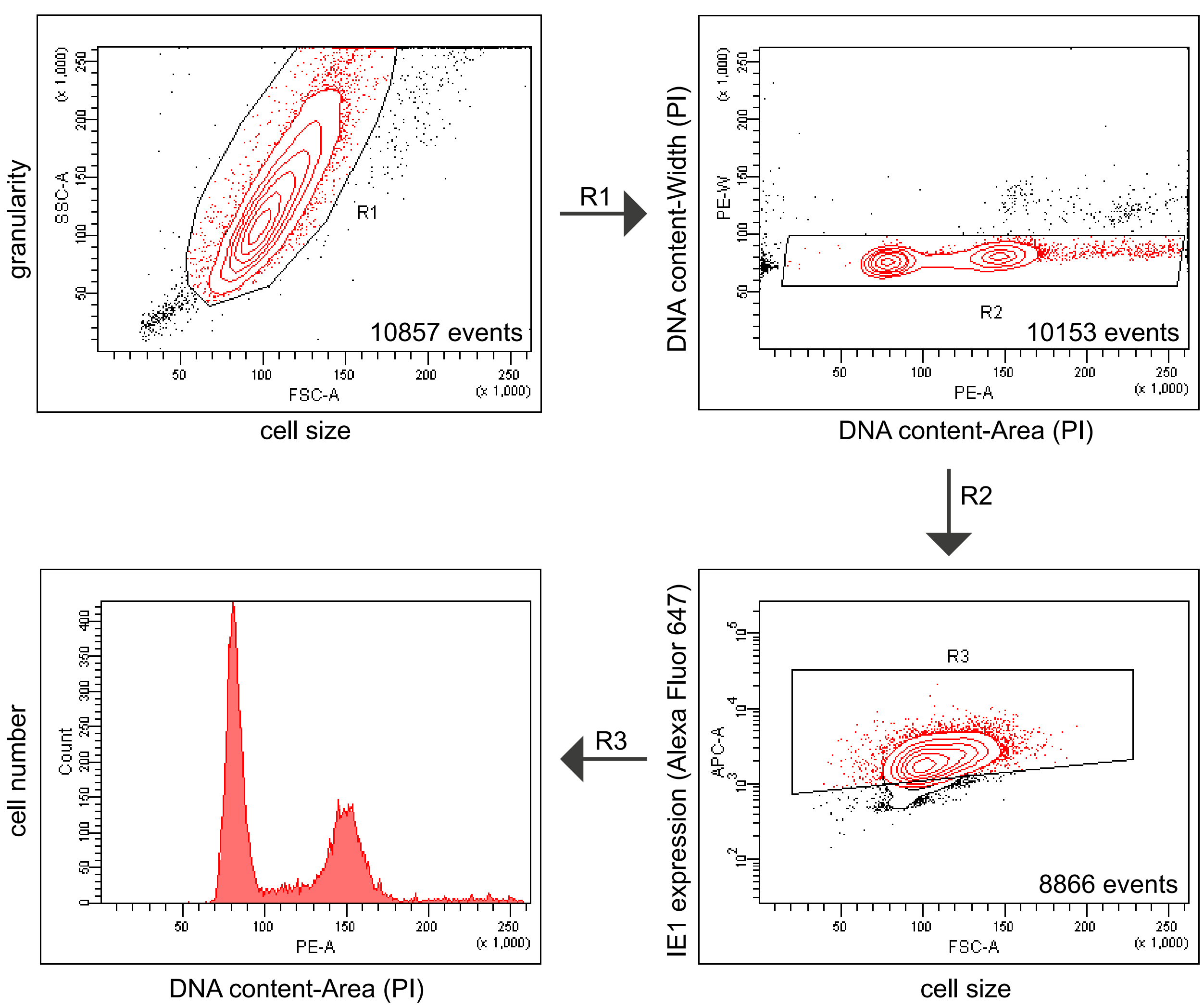

Supplementary Fig. 9. Hierarchical gating strategy for cell cycle analysis of infected cells by flow cytometry. First, a contour plot was created displaying on a linear scale the forward light scatter (FSC) and sideward light scatter (SSC) of measured particles. A region R1 was set that excludes damaged cells and cell aggregates from further analysis. Based on the area (A) and width (W) of the propidium iodide (PI) fluorescence signal (PE channel), a R2 region was defined that excluded cell doublets from further analysis (upper right contour plot). Based on the Alexa Fluor 647 fluorescence (APC channel) of IE1 or M57 immunostaining, a region R3 was set that excludes non-infected and abortively infected cells (lower right contour plot). The P3 population was analyzed for PI fluorescence to create DNA histograms of infected cells. In the case of mock infected cells, DNA histograms include all R2 events. The described gating strategy was used for all data presented in Fig. 5b-c and Supplementary Fig. 10c. Contour levels were set to 15% probability. Outliers falling outside the lowest contour level are displayed as dots.

Supplementary Figure 10

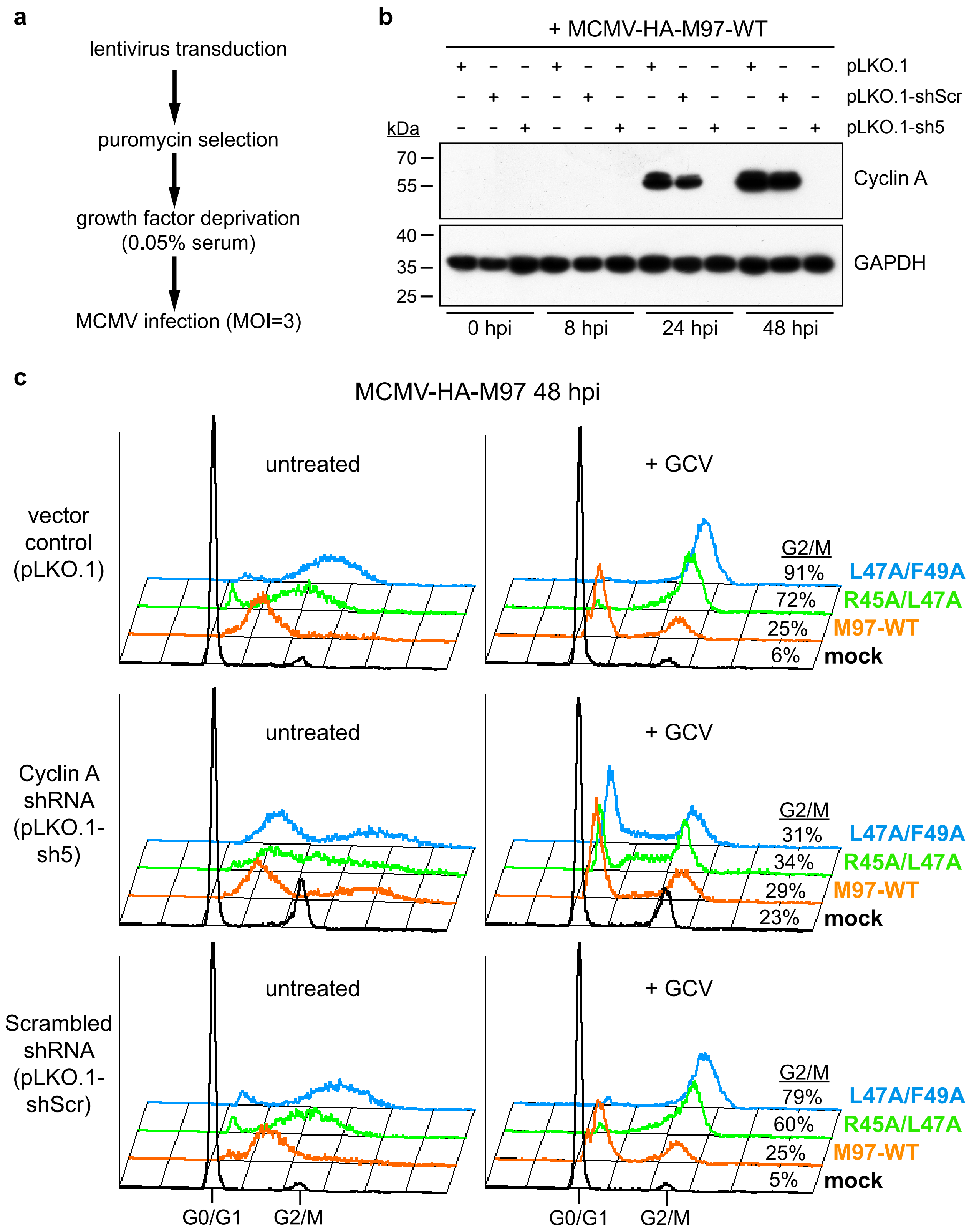

Supplementary Fig. 10. Cyclin A is the key target of M97-RxL/Cy-dependent cell cycle control. (a) Experimental setup. **(b)** Stable lentiviral transduction of the Cyclin A-specific small hairpin RNA (shRNA) sh5 but not of scrambled shRNA (shScr) or the empty shRNA expression vector (pLKO.1) prevents the induction of Cyclin A protein expression by MCMV. **(c)** At 48 h post infection (hpi) with the indicated MCMV variants the DNA content of untreated (left panel) and ganciclovir (GCV) treated cells was analyzed by flow cytometry. Displayed are DNA histograms and the percentages of cells with a G2/M DNA content.

Supplementary Figure 11

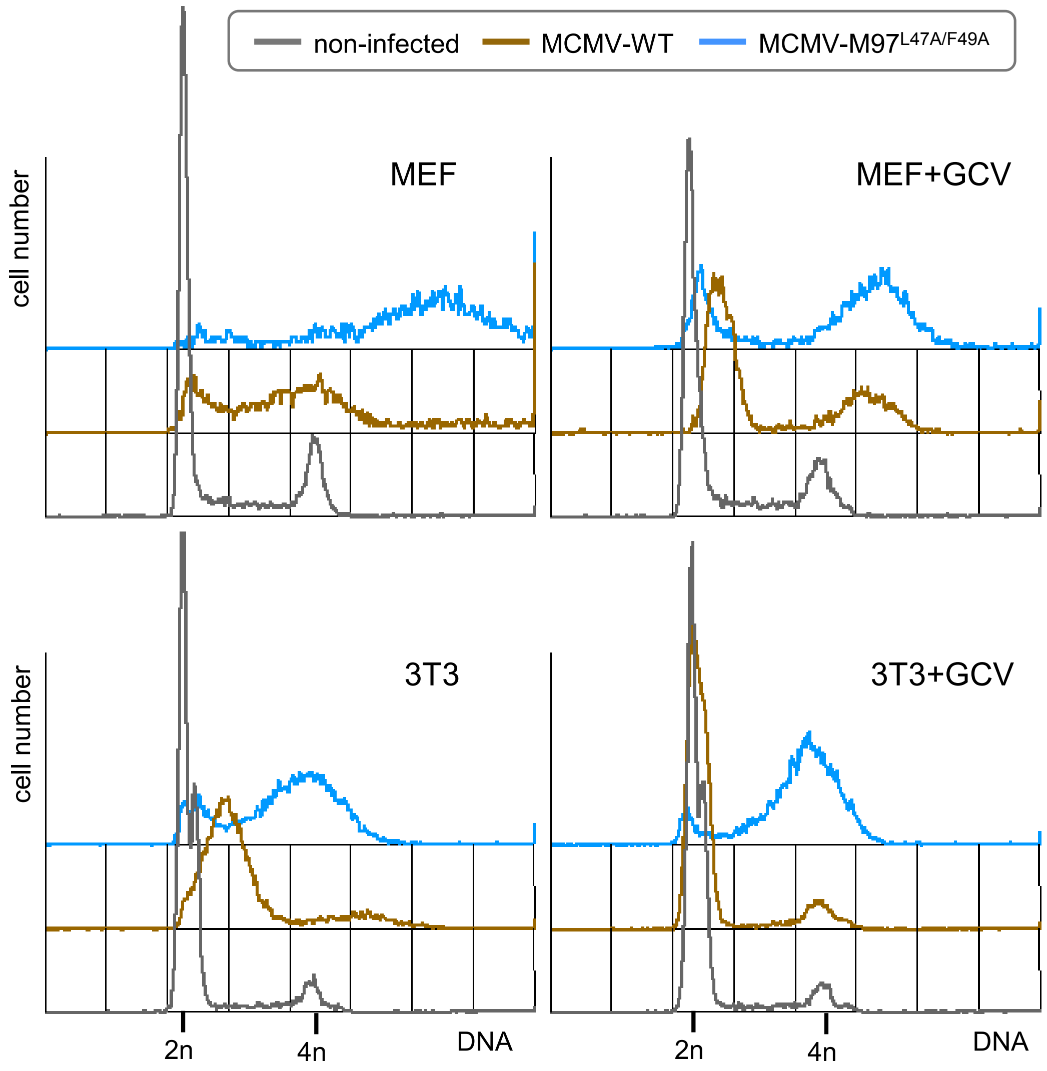

Supplementary Fig. 11. Loss of M97-Cyclin A interaction leads to unscheduled cellular DNA synthesis and over-replication in primary cells. Mouse embryonic fibroblasts (MEF) or NIH-3T3 fibroblasts were synchronized in G0/G1 phase by growth factor deprivation (0.05% serum for 2 days). Cells were infected with M97-RXL/Cy mutant MCMV or the parental WT virus (MOI=2). After removal of the virus inoculum, low serum conditions were maintained. Where indicated, 50 µM ganciclovir (GCV) was added to the culture medium. At 48 h post infection, cells were harvested and analyzed by flow cytometry for DNA content and viral infection markers (IE1 for GCV-treated cells, M57 for untreated cells). DNA histograms of infected and non-infected cell populations are shown.

Supplementary Figure 12

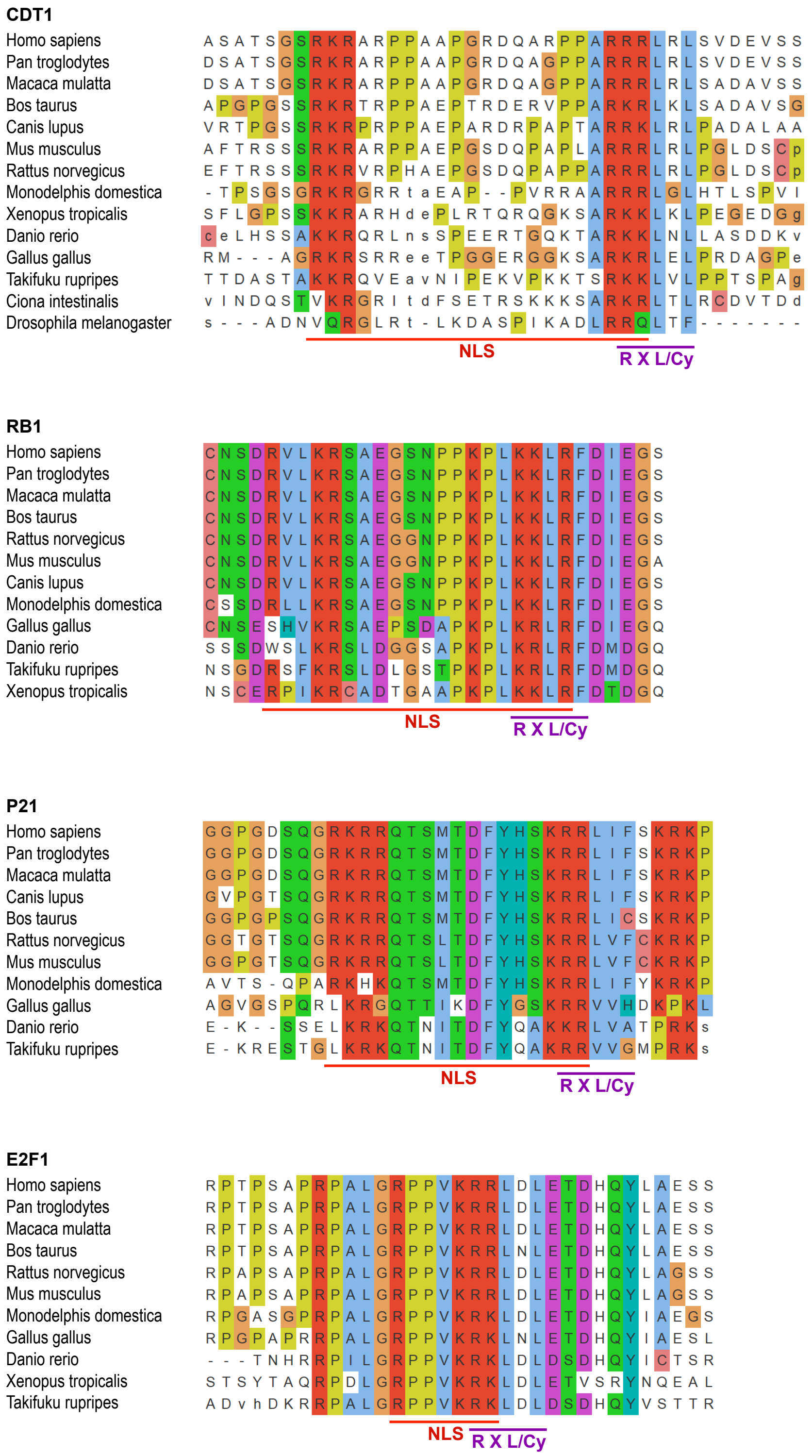

Supplementary Fig. 12. Overlapping NLS-RxL/Cy sequence motifs are conserved features of some key cell cycle regulators. Multiple sequence alignments of NLS-RxL/Cy containing sequences from animal homologues of CDT1, RB1, P21 and E2F1 were generated using the web-based visualization tool ProViz.

SI Methods

Plasmids. The CCAR2 expression plasmid pcDNA-Myc-DBC1 was a gift from Osamu Hiraike (Addgene plasmid #35096)[1]. pEGFPC1-SPOP was a gift from Aimin Liu (Addgene plasmid #128872). Flag-TRIM28 was a gift from Michelle Barton (Addgene plasmid #124960)[2]. pCIneoGFP-BMI1 was a gift from Yutaka Hata (Addgene plasmid #128328). The pLKO.1 - TRC cloning vector was a gift from David Root (Addgene plasmid #10878)[3]. To generate pLKO.1-sh5[4], the oligonucleotides CCNA2-sh5-fw (5'-CCGGGCTTCGAAGTTTGAAGAAATACTCGAGTATTTCTTCAAACTTCGAAGCTTTTTG-3') and CCNA2-sh5-rv (5'-AATTCAAAAAGCTTCGAAGTTTGAAGAAATACTCGAGTATTTCTTCAAACTTCGAAGC-3') were annealed and ligated into the AgeI/EcoRI-digested pLKO.1 backbone. The plasmids psPAX2 and pMDM2.G were gifts from Didier Trono (Addgene plasmids #12260 and #12259).

Lentivirus transduction. For producing recombinant lentiviruses, the pLKO.1 constructs were co-transfected with psPAX2 and pMD2.G into HEK-293T cells. At 2 days post transfection, virus containing cell culture supernatants were harvested and clarified by ultrafiltration. Lentivirus infection of NIH-3T3 cells was performed overnight. The following day, cells were re-plated at lower density and subjected to puromycin (4 µg/ml) selection. Stably transduced cell pools were used for experiments.

Antibodies. For immunoblot analysis, the following antibodies were used: Anti-Myc (clone 4A6, Sigma Aldrich), anti-GFP (ab290, rabbit polyclonal, Abcam), anti-Flag (clone M2, Sigma Aldrich), anti-HA (clone 3F10, Sigma Aldrich). All antibodies were diluted to 1 µg per ml.

Quantitative imaging. For quantitative imaging of immunoblots, a Fusion FX system from Vilber Lourmat was used according to the manufacturer's instructions.

SI References

1. Hiraike, H. *et al.* Identification of DBC1 as a transcriptional repressor for BRCA1. *Br. J. Cancer* **102**, 1061–1067 (2010).
2. Li, J. *et al.* TRIM28 interacts with EZH2 and SWI/SNF to activate genes that promote mammosphere formation. *Oncogene* **36**, 2991–3001 (2017).
3. Moffat, J. *et al.* A Lentiviral RNAi Library for Human and Mouse Genes Applied to an Arrayed Viral High-Content Screen. *Cell* vol. 124 1283–1298 (2006).
4. Ludwig, L. S. *et al.* Genome-wide association study follow-up identifies cyclin A2 as a regulator of the transition through cytokinesis during terminal erythropoiesis. *American Journal of Hematology* vol. 90 386–391 (2015).
