# Supplementary material for: Cross-regulation of viral kinases with cyclin A secures shutoff of host DNA synthesis
Source: Nat Commun. 2020 Sep 24;11:4845. doi: 10.1038/s41467-020-18542-1 (PMC7518283; doi:10.1038/s41467-020-18542-1)
Supplement: Supplementary file 3 — Description of Additional Supplementary Files [file 41467_2020_18542_MOESM3_ESM.docx]

**Description of Additional Supplementary Files**

File Name: Supplementary Data 1
Description: (Sheet 1) Proteins significantly enriched in precipitates of herpesviral kinases. Fold-changes of individual experiments, averages and t-test p values are given. Relates to the heatmap in Figure 1. (Sheet 2) All proteins quantified. Fold-changes of individual experiments, averages and t-test p values are given.

File Name: Supplementary Data 2
Description: SILAC ratios of proteins identified in precipitates of herpesviral kinases pUL97 and BGLF4. Replicate ratios and average ratios are given.

File Name: Supplementary Data 3
Description: (Sheet 1) Significantly enriched proteins in HA-M97-AP at 12 h and 36 h post MCMV infection. (Sheet 2) All proteins identified in SILAC AP-MS experiments of HA-M97 in MCMV infected cells. SILAC ratios of individual replicates, average SILAC ratios, corresponding t-test p-values and iBAQs are given.

File Name: Supplementary Data 4
Description: Results from MS-analysis of whole cell lysate or phosphopeptide enrichment as indicated. Log2 SILAC fold-changes of phosphosites and corresponding proteins are given. Phosphosites were further annotated according to their subcellular localization and their sequence context.

File Name: Supplementary Data 5
Description: Primer sequences. Oligonucleotide primers used for cloning and mutagenesis of plasmids and recombinant viruses.
