# Supplementary material for: Cross-regulation of viral kinases with cyclin A secures shutoff of host DNA synthesis
Source: Nat Commun. 2020 Sep 24;11:4845. doi: 10.1038/s41467-020-18542-1 (PMC7518283; doi:10.1038/s41467-020-18542-1)
Supplement: Supplementary file 9 — Reporting Summary [file 41467_2020_18542_MOESM9_ESM.pdf]

nature research

Corresponding author(s): Lüder Wiebusch

Last updated by author(s): Aug 13, 2020

Reporting Summary

Nature Research wishes to improve the reproducibility of the work that we publish. This form provides structure for consistency and transparency in reporting. For further information on Nature Research policies, see our Editorial Policies and the Editorial Policy Checklist.

Statistics

For all statistical analyses, confirm that the following items are present in the figure legend, table legend, main text, or Methods section.

n/a	Confirmed	
[ ]	[x]	The exact sample size (*n*) for each experimental group/condition, given as a discrete number and unit of measurement
[ ]	[x]	A statement on whether measurements were taken from distinct samples or whether the same sample was measured repeatedly
[ ]	[x]	The statistical test(s) used AND whether they are one- or two-sided<br>*Only common tests should be described solely by name; describe more complex techniques in the Methods section.*
[x]	[ ]	A description of all covariates tested
[ ]	[x]	A description of any assumptions or corrections, such as tests of normality and adjustment for multiple comparisons
[ ]	[x]	A full description of the statistical parameters including central tendency (e.g. means) or other basic estimates (e.g. regression coefficient) AND variation (e.g. standard deviation) or associated estimates of uncertainty (e.g. confidence intervals)
[ ]	[x]	For null hypothesis testing, the test statistic (e.g. *F*, *t*, *r*) with confidence intervals, effect sizes, degrees of freedom and *P* value noted<br>*Give P values as exact values whenever suitable.*
[x]	[ ]	For Bayesian analysis, information on the choice of priors and Markov chain Monte Carlo settings
[x]	[ ]	For hierarchical and complex designs, identification of the appropriate level for tests and full reporting of outcomes
[ ]	[x]	Estimates of effect sizes (e.g. Cohen's *d*, Pearson's *r*), indicating how they were calculated

Our web collection on statistics for biologists contains articles on many of the points above.

Software and code

Policy information about availability of computer code

Data collection	FACSDiva version 6.1.2 (BD Biosciences)<br>NIS-Elements AR version 5.02.01 (Nikon Instruments)<br>Thermo Scientific Xcalibur (Thermo Scientific)
Data analysis	FACSDiva version 6.1.2 (BD Biosciences)<br>CellQuest Pro version 6.0 (BD Biosciences)<br>NIS-Elements AR version 5.02.01 (Nikon Instruments)<br>ImageJ version 1.52e (NIH)<br>Rstudio version 1.2.5033<br>R version 4.0.1.<br>MaxQuant version 1.6.0.1<br>MaxQuant version 1.5.2.8<br>Metascape (www.metascape.org)

For manuscripts utilizing custom algorithms or software that are central to the research but not yet described in published literature, software must be made available to editors and reviewers. We strongly encourage code deposition in a community repository (e.g. GitHub). See the Nature Research guidelines for submitting code & software for further information.

Data

Policy information about availability of data

All manuscripts must include a data availability statement. This statement should provide the following information, where applicable:

- Accession codes, unique identifiers, or web links for publicly available datasets
- A list of figures that have associated raw data
- A description of any restrictions on data availability

MS raw data and MaxQuant output tables have been deposited to the ProteomeXchange Consortium via the PRIDE partner repository with the dataset identifier PXD016334. URL: https://www.ebi.ac.uk/pride/archive/projects/PXD016334. The source data underlying figures 1e, 2b, 2e, 3b-f, 4b, 5a, 5e-f and Supplementary figures 1a-b, 3c-f and 10b are provided as a Source Data file. The source data underlying figures 1b-d, 3a, 4c and Supplementary figures 1a, 1c, 3b, 6b-e, 8b-c are provided in Supplementary Data files 1-4. Fasta files for proteomic searches were downloaded from Uniprot (https://www.uniprot.org/). All other relevant data are available from the corresponding author upon reasonable request.

Field-specific reporting

Please select the one below that is the best fit for your research. If you are not sure, read the appropriate sections before making your selection.

☒ Life sciences ☐ Behavioural & social sciences ☐ Ecological, evolutionary & environmental sciences

For a reference copy of the document with all sections, see nature.com/documents/nr-reporting-summary-flat.pdf

Life sciences study design

All studies must disclose on these points even when the disclosure is negative.

Sample size	No statistical methods were used to predetermine sample size. Sample sizes were chosen based on prior knowledge in the respective experiments and their intrinsic variability as performed in previous studies. Most experiments were performed in triplicates and biological significance was only ascribed when the observed effects were large.
Data exclusions	No data were excluded.
Replication	All experiments analyzed by immunoblot, immunofluorescence, immunoprecipitation and flow cytometry were performed with at least three replicates and representative results are shown. SILAC samples were biological duplicates or triplicates (label-swaps) as indicated in the manuscript. All findings were successfully reproduced in independent experiments.
Randomization	Samples were not randomized as no experimental groups were used in this study.
Blinding	Investigators were not blinded when because collection and analysis of the presented data was not prone to any bias. Furthermore, the readout of many experiments were precise and quantitative measurements (LC-MS/MS, Flow Cytometry) and not based on subjective assessments.

Reporting for specific materials, systems and methods

We require information from authors about some types of materials, experimental systems and methods used in many studies. Here, indicate whether each material, system or method listed is relevant to your study. If you are not sure if a list item applies to your research, read the appropriate section before selecting a response.

Materials & experimental systems

n/a	Involved in the study	
☐	☒	Antibodies
☐	☒	Eukaryotic cell lines
☒	☐	Palaeontology and archaeology
☒	☐	Animals and other organisms
☒	☐	Human research participants
☒	☐	Clinical data
☒	☐	Dual use research of concern

Methods

n/a	Involved in the study	
☒	☐	ChIP-seq
☐	☒	Flow cytometry
☒	☐	MRI-based neuroimaging

Antibodies

Antibodies used	anti-Cyclin A (C19), rabbit polyclonal, sc-596, Santa Cruz Biotechnology<br>anti-Cyclin A (H-432), rabbit polyclonal, sc-751, Santa Cruz Biotechnology<br>anti-cyclin E (M20), rabbit polyclonal, sc-481, Santa Cruz Biotechnology<br>anti-CDK2 (M2), rabbit polyclonal, sc-163, Santa Cruz Biotechnology

anti-Flag, clone M2, mouse IgG1, F1804, Sigma Aldrich
anti-GFP, rabbit polyclonal, ab290, Abcam
anti-HA, clone 3F10, rat IgG1, 11867423001, Sigma Aldrich (Roche)
anti-IE1, clone Croma 101, mouse IgG1, provided by Prof. Jonjic, University of Rijeka
anti-M45, clone M45.01, mouse IgG2a, HR-MCMV-13, Center for Proteomics, University of Rijeka
anti-M57, clone M57.02, mouse IgG2a, HR-MCMV-06, Center for Proteomics, University of Rijeka
anti-M99, mouse antiserum, provided by Dr. Le-Trilling, University of Essen
anti-Myc, clone 4A6, mouse IgG1, 05-724, Sigma Aldrich (Upstate)
Alexa Fluor 488 coupled goat anti-mouse IgG, Thermo Fisher Scientific
Alexa Fluor 647 coupled goat anti-mouse IgG, Thermo Fisher Scientific

Validation

All antibodies used here have been previously reported and are routinely used in CMV and cell cycle studies.
anti-Cyclin A (C19) was validated for Western blot analysis of murine and human cells using Cyclin A-specific shRNA-mediated knockdown (this manuscript, Supplementary Fig. 10b; product information http://datasheets.scbt.com/sc-596.pdf).
anti-Cyclin A (H-432) has been validated for immunoprecipitation from human and murine cell extracts by Western blot analysis of the output material using a different, independently validated Cyclin A (C19) antibody (see Fig. 1e; product information https://datasheets.scbt.com/sc-751.pdf).
anti-cyclin E (M20) has been validated for Western blot analysis of rat and murine cell lines (see procuct information http://datasheets.scbt.com/sc-481.pdf).
anti-CDK2 (M2) has been validated for Western blot analysis of human and mouse cell lines (see product information http://datasheets.scbt.com/sc-163.pdf).
anti-Flag, clone M2, has been validated for Western blot analysis of human cells (see product infromation https://www.sigmaaldrich.com/catalog/product/sigma/f1804?lang=de&region=DE; http://www.molecularinfo.com/MTM/J/J3/FlagAb.pdf).
anti-GFP (ab290) was validated for Western blot analysis of human cells (see product information https://www.abcam.com/gfp-antibody-ab290.html).
anti-HA, clone 3F10, has been validated for Western blot, immunoprecipitation and immunofluorescence analysis of HA-epitope tagged proteins (see product information https://www.sigmaaldrich.com/content/dam/sigma-aldrich/docs/Roche/Bulletin/1/12013819001bul.pdf).
anti-IE1, clone Croma 101, has been validated for analysis of MCMV infected cells by Western blot and flow cytometry (see DOI: 10.1128/JVI.07181-11 and product information https://products.capri.com.hr/product/anti-m123-ie1-mcmv/).
anti-M45, clone M45.01, has been validated for Western blot analysis of MCMV infected cells (see product information https://products.capri.com.hr/product/anti-m45-mcmv/).
anti-M57, clone M57.02, has been validated for Western blot and immunofluorescence analysis of MCMV infected cells (see product information https://products.capri.com.hr/product/anti-m57-mcmv/).
anti-M99, mouse antiserum, has been validated for Western blot analysis of MCMV infected cells (see https://www.ncbi.nlm.nih.gov/pmc/articles/PMC4524224/).
anti-Myc, clone 4A6, has been validated for Western blot analysis of human cells (see product information https://www.sigmaaldrich.com/catalog/product/mm/05724?lang=de&region=DE).

Eukaryotic cell lines

Policy information about cell lines

Cell line source(s)	HEK-293T were obtained from American Type Culture Collection (ATCC® CRL-3216™).<br>NIH-3T3 were obtained from Cell Lines Service (CLC item number 400101).
Authentication	Cell lines were not further authenticated
Mycoplasma contamination	All cell lines were tested negative for mycoplasma contamination.
Commonly misidentified lines<br>(See ICLAC register)	No commonly misidentified cell lines were used.

Flow Cytometry

Plots

Confirm that:

- [x] The axis labels state the marker and fluorochrome used (e.g. CD4-FITC).
- [x] The axis scales are clearly visible. Include numbers along axes only for bottom left plot of group (a 'group' is an analysis of identical markers).
- [x] All plots are contour plots with outliers or pseudocolor plots.
- [x] A numerical value for number of cells or percentage (with statistics) is provided.

Methodology

Sample preparation

The cell culture supernatant containing floating cells was pipetted into a polypropylene centrifuge tube. The remaining adherent cells were washed with PBS and then detached from the plastic surface of the culture dish by incubation in Trypsin-

	EDTA. The resulting cell suspension was combined with the culture supernatant in the centrifuge tubes. Fetal calf serum was added at 10% final concentration to inactivate Trypsin. Cells were collected by centrifugation in a swing-out rotor at 300 g. After removal of the supernatant, the cell pellet was resuspended in 1 ml PBS. 3.5 ml of absolute ice-cold ethanol were added dropwise while vortexing the cell suspension at medium strength. Subsequently, samples were incubated for at least 16 h on ice. Next, cells were pelleted by centrifugation at 400 g and washed once with PBS. Then, the cells were incubated with one of the following primary antibodies diluted in PBS/1%BSA on ice for 12-16 h: anti-IE1 (clone Croma 101) or anti M57 (clone m57.02). After washing once with PBS/1%BSA, cells were incubated in Alexa Fluor 647-coniugated anti-mouse IgG/PBS/1% BSA for 1-2 h at 25°C. Cells were washed again in PBS/1%BSA and resuspended in PBS/ 0.1 mg/ml RNAse A/25 µg/ml propidium iodide (PI). After 15 min incubation a 25°C, cells were ready for flow cytometry.
Instrument	FACSCanto II flow cytometer (BD Biosciences)
Software	FACSDiva (BD Biosciences)<br>CellQuest Pro (BD Biosciences)<br>ModFit LT (Verity Software House)
Cell population abundance	For determination of cellular DNA content by flow cytometry, only intact single cells were analyzed. Acquisition was stopped automatically when 10,000 events have fulfilled the respective gating criteria (see below). In the case of MCMV infected cells, only cells staining positive for the viral antigene IE1 were considered. Based on IE1 expression, the purity of the analyzed cell populations was very high. The high sample purity was facilitated by a very synchronous infection, resulting from cell cycle synchronization before infection and from the high multiplicity of infection used.
Gating strategy	A dot plot was created displaying on a linear scale the forward light scatter (FSC) and sideward light scatter (SSC) of measured particles. An FSC threshold was set for exclusion of cell debris. A region P1 was set that excludes larger cell debris, cell doublets and aggregates from further analysis. Based on the area (A) and width (W) of the propidium iodide fluorescence signal (PE channel), a P2 region was defined that excluded doublets of G1 cells from further analysis. Finally, based on the Alexa Fluor 647 fluorescence (APC channel) of the IE1/M57 immunostaining, a region P3 was set that selects MCMV infected cells and excludes non-infected and abortively infected cells. The P3 population was analyzed for propidium iodide fluorescence to create DNA histograms/cell cycle profiles of infected cells. In the case of mock infected cells, DNA histograms included all P2 events.

☒ Tick this box to confirm that a figure exemplifying the gating strategy is provided in the Supplementary Information.
